# Long-term proton pump inhibitor use is a risk factor for mortality in patients hospitalized for COVID-19

**DOI:** 10.3906/sag-2103-80

**Published:** 2021-08-30

**Authors:** Ahmet YOZGAT, Benan KASAPOĞLU, Güray CAN, Alpaslan TANOĞLU, Yusuf Serdar SAKİN, Kadir Serkan YALÇIN, Müjgan GÜRLER, Mustafa KAPLAN, Mehmet Göktürk KABAN, Mehmet KIRSOY, Umut KARA, Murat KEKİLLİ

**Affiliations:** 1 Department of Gastroenterology, Faculty of Medicine, Ufuk University, Ankara Turkey; 2 Department of Gastroenterology, Faculty of Medicine, Lokman Hekim University, Ankara Turkey; 3 Department of Gastroenterology, Faculty of Medicine, Abant İzzet Baysal University, Bolu Turkey; 4 Department of Gastroenterology, Haydarpaşa Training Hospital, University of Health Sciences, İstanbul Turkey; 5 Department of Gastroenterology, Gülhane Training and Research Hospital, University of Health Sciences, Ankara Turkey; 6 Department of Internal Medicine, Faculty of Medicine, Lokman Hekim University, Ankara Turkey; 7 Department of Internal Medicine, Faculty of Medicine, Abant İzzet Baysal University, Bolu Turkey; 8 Department of Internal Medicine, Haydarpaşa Training Hospital, University of Health Sciences, İstanbul Turkey; 9 Department of Anesthesiology and Reanimation, Gülhane Training And Research Hospital, University of Health Sciences, Ankara Turkey; 10 Department of Gastroenterology, Faculty of Medicine, Gazi University, Ankara Turkey

**Keywords:** Covid-19, proton pump inhibitors, mortality

## Abstract

**Background and aim:**

The aim of this study is to evaluate whether the long-term (≥4 weeks) use of proton pump inhibitors (PPIs) is a risk factor for intubation requirement and mortality in patients hospitalized for COVID-19.

**Materials and methods:**

In this multicentric retrospective study, a total of 382 adult patients (≥18 years of age) with confirmed COVID-19 who were hospitalized for treatment were enrolled. The patients were divided into two groups according to the periods during which they used PPIs: the first group included patients who were not on PPI treatment, and the second group included those who have used PPIs for more than 4 weeks

**Results:**

The study participants were grouped according to their PPI usage history over the last 6 months. In total, 291 patients did not use any type of PPI over the last 6 months, and 91 patients used PPIs for more than 4 weeks. Older age (HR: 1.047, 95% CI: 1.026–1.068), current smoking (HR: 2.590, 95% CI: 1.334–5.025), and PPI therapy for more than 4 weeks (HR: 1.83, 95% CI: 1.06–2.41) were found to be independent risk factors for mortality

**Conclusion:**

The results obtained in this study show that using PPIs for more than 4 weeks is associated with negative outcomes for patients with COVID-19. Patients receiving PPI therapy should be evaluated more carefully if they are hospitalized for COVID-19 treatment.

## 1. Introduction

In December 2019, a pneumonia-causing disease was discovered in Wuhan, China, which was later identified as COVID-19 by the World Health Organization (WHO) [1]. COVID-19 spread rapidly throughout the world and was identified as a pandemic by the WHO in March 2020 [2]. The etiological agent of this disease was named severe acute respiratory syndrome coronavirus 2 (SARS-CoV-2) because of its phylogenetic similarity with SARS virus. This disease exhibits a wide spectrum of manifestations, ranging from asymptomatic disease to pneumonia, severe respiratory failure, and even death, and it is generally more severe in the elderly and individuals with comorbid diseases [3]. COVID-19 primarily affects the respiratory system and causes severe pneumonia, with the possibility of ground-glass opacities in the lung and cardiac damage occurring [4].

Proton pump inhibitors (PPIs) fully inhibit the H^+^-K^+^-ATPase pump in parietal cells for a long time and also inhibit basal and stimulated gastric acid secretion for up to 16–18 h [5]. Generally, PPIs are superior to other antisecretory drugs in the presence of gastroesophageal reflux disease, peptic ulcer, and dyspepsia [6]. Since the risk of developing tolerance against the effects of PPIs is low and they remain effective for a long time, they have been increasingly used in the treatment of acid-related diseases and have become the first treatment option [7]. It should, however, be noted that the long-term use of PPIs may cause some problems, such as vitamin and mineral absorption disorders, decreased effectiveness of antiplatelet drugs, increased risk of osteoporotic fractures, and acute and chronic renal failure [8,9]. It has also been found that using PPIs causes changes in the intestinal microbiota and may, therefore, increase the rate of infections due to enteric pathogens, such as infection with *Clostridium difficile* [10]. Moreover, it has been shown that the reduced gastric acidity (hypochlorhydria) resulting from long-term PPI use may cause an increased risk of community-acquired pneumonia due to bacterial colonization and aspirations in the stomach [11]. Therefore, the aim of this study is to evaluate whether the long-term (≥4 weeks) use of PPIs has an effect on the mortality and morbidity of patients who are hospitalized for COVID-19. 

## 2. Materials and methods 

In this multicentric retrospective study, 382 adult patients (≥18 years of age) with confirmed COVID-19 who were hospitalized for treatment were enrolled. This study was approved by the Local Ethics Committee of Lokman Hekim University, Ankara, Turkey.

Diagnosis of COVID-19 was confirmed using nasopharyngeal swab real-time reverse transcriptase PCR according to the WHO guidelines^1^Organization WH. Assessment tool for laboratories implementing COVID-19 virus testing: interim guidance . Website. https://apps.who.int/iris/handle/10665/331714 [acessed on ].. Patients were treated in line with the recommendations of the Turkish Health Ministry COVID-19 adult patient treatment guidelines^2^Bakanligi TCS. COVID-19 (SARS-CoV-2 ENFEKSİYONU) ERİŞKİN HASTA TEDAVİSİ. Website https://covid19.saglik.gov.tr/TR-66926/eriskin-hasta-tedavisi.html [acessed on ].. Demographic features, complete medical history, and the laboratory findings of the study participants at admission were obtained from the medical records. Patients under any other type of antisecretory medication (H2 receptor antagonists) were not included in the study. Patients with missing data (n = 21), patients with any known malignancies (n = 5), patients under immunosuppressive therapy (n = 4), and patients under 18 years of age (n = 2) were also excluded from the study. In total, 411 patients were evaluated for the study, 29 of whom were excluded according to the exclusion criteria, ultimately leading to the inclusion of 382 patients.

Some data in the literature have supported the notion that using PPIs increases the risk of pneumonia during the first month of therapy. Therefore, we determined the cutoff for the use of PPIs as 4 weeks [12]. Patients were divided into two groups according to the periods during which they used PPIs: the first group included those who were not on PPI treatment, and the second group included those who have used PPIs for more than 4 weeks. Patients taking any type of PPIs from time to time were not included in the study. 

Age, sex, the presence of comorbid diseases, medications used, duration of drug use, smoking habits, treatments received after the diagnosis of the disease, whether these patients were admitted to the intensive care unit or not, and whether they were intubated were recorded with their outcomes. Those whose treatment or hospitalization periods were still ongoing during the data collection were not included in the study.

### 2.1. Statistical analysis 

All statistical analyses were performed using IBM SPSS Statistics v. 22.0 (IBM Corp., Armonk, NY, USA). Independent student’s t-test and one-way analysis of variance (ANOVA) were used for continuous variables, and the results are presented as the mean ± standard deviation. The chi-square test was used for categorical variables in independent groups, and the results are presented as numbers and percentages. Normal distribution for continuous variables was evaluated with the Kolmogorov–Smirnov test. The Mann–Whitney U test was used to perform a comparative analysis between the two independent groups. Cox regression analysis was used to evaluate the risk factors related to intubation and mortality. A p-value of 0.05 was regarded as statistically significant.

## 3. Results

A total of 382 patients were included in this study and divided into two groups according to their PPI usage history over the last 6 months: 291 patients reported that they did not use any type of PPI over the last 6 months, and 91 patients reported using PPIs for more than 4 weeks (lansoprazole, n = 12; esomeprazole, n = 10; pantoprazole, n = 58; and rabeprazole, n = 11). 

Table 1 summarizes the demographic characteristics of the study participants as well as their patterns of use of PPIs. Those who reported using PPIs for more than 4 weeks were found to be significantly older (p = 0.001). Regarding the presence of comorbid chronic diseases (e.g., hypertension, diabetes, asthma, or chronic obstructive pulmonary disease), hypertension, and diabetes were found to be significantly more common in the PPI-using group (Table 1). No significant difference was found between groups regarding the distribution of medications used for the treatment of COVID-19 (p > 0.05). 

**Table 1:** Demographic characteristics according to PPI therapy.

Parameters	PPI Therapy		p
	No current PPI use (G1)n=291	>4 weeks PPI use (G2)n=91	
Age, yearsMedian (IQR)	49 (32-66)	70 (59-77)	<0.001*
Sex, n (%)Female	111 (38.1)	32 (35.2)	0.6**
Male	180 (61.9)	59 (64.8)	
Current smoking status, n (%)No	219 (75.3)	50 (54.9)	<0.001**
Yes	72 (24.7)	41 (45.1)	
Comorbidities, n (%)No	160 (55)	12 (13.2)	<0.001**
Yes	131 (45)	79 (86.8)	
Hypertension, n (%)No	187 (67.8)	36 (39.6)	<0.001**
Yes	89 (32.2)	55 (60.4)	
ACE inh or ARB Therapy, n (%)No	211 (78.7)	45 (50.6)	<0.001**
Yes	57 (21.3)	44 (49.4)	
Diabetes mellitus, n (%)No	226 (82.8)	64 (71.9)	0.026**
Yes	47 (17.2)	25 (28.1)	
Antidiabetic Therapy, n (%)No	238 (88.1)	64 (72.7)	0.001**
Yes	32 (11.9)	24 (27.3)	
Place of treatment, n (%)Pandemic service	245 (84.2)	54 (59.3)	<0.001**
Intensive care unit	46 (15.8)	37 (40.7)	
Intubation, n (%)No (IQR)	254 (87.3)	52 (57.1)	<0.001**
Yes	37 (12.7)	39 (42.9)	
Exitus, n (%)No	254 (87.3)	57 (62.6)	<0.001**
Yes	37 (12.7)	34 (37.4)	
Duration of stay in hospital, dayMedian (IQR)	14 (8-14)	13 (7-18)	0.298*
Duration of stay in ICU, dayMedian (IQR)	8 (4-13)	8 (5-20)	0.259*

*Mann–Whitney U test; **Chi-Square test; PPI: proton pump inhibitor; ACE inh: angiotensin converting enzyme inhibitor; ARB: angiotensin receptor blocker; ICU: intensive care unit.

Table 2 summarizes the results of the Cox regression analysis for intubation and mortality. According to the Cox regression analysis, current smoking, the use of an angiotensin converting enzyme (ACE) inhibitor or angiotensin receptor blocker (ARB), and PPI therapy for more than 4 weeks were found to be independent risk factors for intubation. In addition, older age (HR: 1.047, 95% CI: 1.026–1.068), current smoking (HR: 2.590, 95% CI: 1.334–5.025), and PPI therapy for more than 4 weeks (HR: 1.83, 95% CI: 1.06–2.41) were found to be independent risk factors for mortality. 

**Table 2:** The evaluation of risk factors related to intubation and mortality with regression analyses (n = 382).

	Risk factors related to intubation				Risk factors related to mortality			
	Univariate logistic regression (LR) analysis		Multivariate LR model		Univariate COX regression(CR) analysis		Multivariate CR model	
	Crude OR (95%CI)	P	Adjusted OR (95% CI)	P	Crude HR (95% CI)	P	Adjusted HR (95% CI)	P
Sex (ref: male)	1.114 (0.665–1.865)	0.682	3.022 (1.183–7.719)	0.021	1.418 (0.882–2.281)	0.150	1.533 (0.802)	0.196
Age	1.056 (1.039–1.074)	<0.001	1.039 (1.014–1.065)	0.002	1.043 (1.028–1.059)	<0.001	1.047 (1.026–1.068)	<0.001
Current smoking* (ref: no)	4.723 (2.784–8.011)	<0.001	9.364 (3.769–23.267)	<0.001	2.011 (1.231–3.284)	0.005	2.590 (1.334–5.025)	0.005
Hypertension(ref: no)	6.633 (3.681–11.953)	<0.001	0.497 (0.156–1.584)	0.237	3.187 (1.859–5.464)	<0.001	0.937 (0.400–2.192)	0.880
Diabetes mellitus (ref: no)	2.696 (1.492–4.873)	0.001	0.785 (0.351–1.756)	0.556	2.107 (1.250–3.551)	0.005	1.271 (0.719–2.246)	0.409
ACE inh or ARB use(ref: no use)	8.992 (4.963–16.293)	<0.001	5.363 (1.870–15.382)	0.002	2.566 (1.529–4.305)	<0.001	0.937 (0.433–2.028)	0.870
PPI therapy, ≥4 weeks PPI use(ref: no use)	5.149 (3.001–8.833)	<0.001	4.532 (2.233–9.200)	<0.001	1.944 (1.188–3.182)	0.008	1.83 (1.065–2.419)	0.031

PPI: proton pump inhibitor; ACE inh: angiotensin converting enzyme inhibitor; ARB: angiotensin receptor blocker; OR: odds ratio; HR: hazard ratio; CI: confidence interval.

## 4. Discussion 

The results obtained in this study show that using PPIs for more than 4 weeks is an independent risk factor for both intubation and mortality. For patients with COVID-19, which is currently affecting millions of people worldwide and is causing high rates of mortality, it is critical to determine the risk factors that may lead to intubation requirement and mortality.

In general, PPIs are one of the most frequently prescribed drugs worldwide. Long-term PPI use has been related to some adverse events associated with vitamin absorption and alterations in intestinal microbiota [13]. These effects are associated with hypochlorhydria, which disturbs the body’s defenses against ingested viruses and bacteria [14]. 

Data regarding the effects of long-term PPI use on the outcomes of patients diagnosed with COVID-19 have rapidly increased. For example, recently, Almanaro et al. found a dose–response relationship between COVID-19 positivity and PPI use, and they found that patients who take PPIs twice a day are at an increased risk for testing positive for COVID-19 on PCR [15]. In general, the normal pH level is 3 or less in a healthy stomach. While this acidic environment impairs the infectivity of SARS-CoV-1, the virus responsible for severe acute respiratory syndrome, the higher pH resulting from the use of PPIs does not inactivate the virus [16].

SARS-CoV-2 can enter the body via the gastrointestinal (GI) system through the angiotensin-converting enzyme 2 receptor, which is widely expressed in the GI system [17,18]. This helps the virus spread easily outside the GI system and cause inflammation in other systems [19]. The gut microbiota plays an essential role in the regulation of metabolic, defensive, and immunological processes in the human body. Any change in the microbial balance, namely, dysbiosis, can directly affect the immunological response [20–22].PPIs cause hypoacidity, which results in negative effects on the stomach functions and defense mechanisms of the body, leading to decreased gastric mucus viscosity, delayed gastric emptying, and increased bacterial translocation and bacterial load [23]. Impairment of neutrophil functions induced by PPI use can impair the body’s ability to recover from an infection and may contribute to harmful outcomes for an infection [24]. In this respect, our results reporting the negative effects of PPI use for more than 4 weeks on intubation requirement and mortality rates of patients with COVID-19 may be associated with the altered immunity and intestinal microbiota of the patients using PPIs. 

Several recently published studies have shown that using PPIs is associated with poor outcomes for patients with COVID-19. In a national cohort study by Lee et al., the authors found that using PPIs is associated with mortality (adjusted OR: 1.79, 95% CI: 1.3–3.1) and poor clinical outcomes. However, since these data have been taken from electronic health records, they may not match the actual usage data [25]. In another study, Ramachandran et al. showed that, in 295 patients hospitalized for COVID-19, prehospitalization PPI exposure is an independent risk factor for mortality (OR: 2.33, 95% CI: 1.18–4.59) [26]. It has also been shown that H2 receptor blockers do not increase but rather decrease the risk of mortality for patients with COVID-19 [15,27]. In a pooled meta-analysis, Hariyanto et al.[28] also showed that using PPIs is associated with a significant increase in the risk of severe COVID-19. However, in all of these reports, data on the duration or type of PPIs used were not available or standardized. In contrast to these findings, in an opinion paper evaluating the data of many studies, the potential benefits of using PPIs in the treatment of COVID-19 were evaluated, and it was stated that PPIs may be effective in the treatment of COVID-19 [29].

This study has several limitations, the most important of which are the small number of patients included and its retrospective design. All the data were obtained from hospital records and patient databases. We did not evaluate the presence of pneumonia in our patients, which is considered another limitation of this study. Therefore, we were unable to suggest a direct association between PPI use and pneumonia. Although our two groups were not balanced in terms of age, smoking habits, and the presence of comorbidities, this did not affect the results thanks to the significant findings obtained in the regression analysis. Because of the highly common use of these medications, larger prospective, randomized, controlled studies are warranted to determine the direct effects of long-term PPI use on the outcomes of patients with COVID-19.

In conclusion, we found that using PPIs for more than 4 weeks is associated with negative outcomes for patients with COVID-19. Therefore, patients under PPI therapy should be evaluated more carefully if they are hospitalized for COVID-19.

## Author contributions

A.Y: conceptualization, formal analysis, investigation, Writing; B.K: conceptualization, formal analysis, investigation, writing; G.C: data curation, software, validation, writing ; A.T: data curation, formal analysis, writing; Y.S.S: data curation, formal analysis, writing; K.S.Y: data curation, investigation, software, validation; M.G: data curation, investigation, software, validation; M.K: data curation, formal analysis, writing; M.G.K: data curation, investigation, software; M.K: data curation, formal analysis, writing , U.K: data curation, formal analysis, writing; M.K: conceptualization, formal analysis, Investigation, writing.

## Ethical approval/Informed consent

Lokman Hekim University Local Ethics Committee approved the study with the number of 2021-023. Informed consent was not obtained from the patients because the study was retrospective.
